# Temporal Trends of Common Female Malignances on Breast, Cervical, and Ovarian Cancer Mortality in Japan, Republic of Korea, and Singapore: Application of the Age-Period-Cohort Model

**DOI:** 10.1155/2018/5307459

**Published:** 2018-03-21

**Authors:** Jinyao Wang, Haizhen Lv, Zhilin Xue, Lu Wang, Zhiqiang Bai

**Affiliations:** ^1^Department of Preventive Medicine, School of Medicine, Shanxi Datong University, Xingyun Road, Datong 037009, China; ^2^Department of Basic Medicine, School of Medicine, Shanxi Datong University, Xingyun Road, Datong 037009, China; ^3^Department of Epidemiology and Biostatistics, School of Public Health, Wuhan University, 115 Donghu Road, Wuhan 430071, China; ^4^School of Life Sciences, Shanxi Datong University, Xingyun Road, Datong 037009, China

## Abstract

**Background:**

Reproductive system cancer is an important cause of morbidity and mortality worldwide which threatens women's health and lives. Breast, cervical, and ovarian cancer have the higher incidence and mortality among a series of gynecology malignant tumor. We aimed to compare and assess the temporal trends of common female malignances on breast, cervical, and ovarian cancer mortality in developed regions of Asia including Japan, Republic of Korea, and Singapore and analyze the detached effects of chronological age, time period, and birth cohort by age-period-cohort (APC) analysis.

**Methods:**

The mortality data for these three cancers were collected from the WHO Mortality Database in Japan, Republic of Korea, and Singapore from 1954 to 2013, from 1989 to 2013, and from 1964 to 2013, respectively. We fitted an age-period-cohort model and intrinsic estimator method to estimate the independent effect of each age, time period, and birth cohort on cancer mortality and describe the secular changes in three Asian countries.

**Results:**

For the overall trends of breast cancer, the ASMRs of breast cancer showed a general increasing trend among three countries during the study periods while the change pattern in Singapore was different from the rest of the two countries for cervical and ovarian cancer. By APC analysis, the three cancer mortality risks generally increased with age and decreased with birth cohort. For period effects of breast and ovarian cancer, increasing effects with time were observed; however, for period effects of cervical cancer, converse change pattern was presented among three countries.

**Conclusions:**

Our study shows that the ASMRs of breast, cervical, and ovarian cancer remain high in Singapore compared to Japan and Korea. Generally speaking, the mortality risk of three cancers increased with age, and period and cohort effects may collectively affect the common female malignances mortality for East Asian women.

## 1. Introduction

Breast, cervical, and ovarian cancer are the most common reproductive cancers occurring in female population. Breast cancer is the leading cancer in women, with approximately 1.67 million new diagnosed cases worldwide in 2012 alone. According to a report by Globocan, breast cancer incidence has increased by more than 20%, while mortality has increased by 14% since 2008 [1]. Cervical cancer is the fourth most common cancer in women and the seventh overall, and almost nine out of ten (87%) cervical cancer deaths occur in the less developed regions. Furthermore, it is estimated that there were 266,000 deaths from cervical cancer worldwide in 2012, accounting for 7.5% of all female cancer deaths [2]. Ovarian cancer is a common gynecologic malignancy which significantly threatens women's lives. The ovarian cancer has high morbidity and mortality rates among cancers of the reproductive system. According to the global estimates more than 0.22 million patients are newly diagnosed yearly and approximately 0.14 million people die from this disease each year [3].

Significant differences in the incidence and mortality of female breast cancer were presented throughout the whole world. The United States and Northern Europe have the highest incidence of female breast cancer while the Asian countries have the lowest incidence of it. However, the gap has been reduced with the improvement of living standard and the change of life style, which presented the increasing secular trend of breast cancer mortality in Asian, especially in Japan and Singapore [4]. Cervical cancer is a worldwide problem which accounts for 9.8% of all new cases worldwide. The geographical distribution of this cancer is uneven which ranks as 2nd in developing countries, with a relative frequency of 15% of all cancers in women, whereas it ranks as 5th in developed countries, with a relative frequency of 4.4%. Even in high-income regions, there has been little improvement in survival rates of this disease [5, 6]. Ovarian cancer is the leading cause of mortality in developed countries from the gynecological malignancies in women of all ages [7]. The incidence and mortality of this disease vary a lot in different regions of the world which have the highest incidence in Europe and the lowest rates in Asia and Africa [8].

The age-period-cohort (APC) model is a useful tool which has long been used in statistical analysis of human populations. It has been widely utilized in fields of demography and epidemiology to assess the effects of the three factors in disease incidence or mortality rates [9]. Age, period (year of death), and cohort (year of birth) are three independent factors which affect the incidence and mortality trends of disease and the model of APC can detach the independent effects of chronological age, time period, and birth cohort with the considering of three factors simultaneously [10]. The age effects represent the different risks associated with different age groups. The period effects represent factors that affect all age groups simultaneously, owing to changes in screening practices, diagnostic methods, and disease classification, among other factors. The cohort effects describe the temporal variations in rates among groups born during the same period and which reflect changes in lifestyle and exposure to risk factors in different generations [9, 11].

Investigations of long-term trends on cancer incidence and mortality rates can explore the etiology of the illness. Despite the fact that a number of studies have focused on the epidemiology of various common female malignances worldwide, the secular mortality trends of breast, cervical, and ovarian cancer among Asian women in developed areas are less reported. In our study, in order to explore the changing patterns of common female reproductive cancers in developed regions of Asian, we examined three countries including Japan (1954–2013), Republic of Korea (1989–2013), and Singapore (1964–2013) from the database of WHO Mortality using an age-period-cohort analysis.

## 2. Materials and Methods

### 2.1. Data Source

All research data of breast, cervical, and ovarian cancer mortality used in this study were extracted from the WHO Mortality Database in three countries including Japan, Republic of Korea, and Singapore (http://www-dep.iarc.fr/WHOdb/WHOdb.htm). To better compare and analyze the temporal trends of common female malignances on breast, cervical, and ovarian cancer mortality in developed regions of Asia, we selected three countries including Japan, Republic of Korea, and Singapore during the period of 1954–2013, 1989–2013, and 1964–2013, respectively. In our study, for the reason that the incidence and mortality of ovarian cancer were more lower than another two cancers, most countries throughout the world may not pay enough attention to this disease and the data for ovarian provided by various countries may not reach the standard of the International Association for Cancer Registration (IARC) until 1994; thus the mortality data of ovarian cancer for the three countries was limited and only available during a short period; therefore, data for the period 1994–2013 were collected from the WHO Mortality Database. Moreover, the mortality data on ovarian cancer in 1994 of Republic of Korea is not available, so we calculated the average of the nearest four calendar years instead of the average value from 1994 to 1998 [12]. According to the International Classification of Diseases of the 10th revision (ICD-10), breast cancer, cervical cancer, and ovarian cancer were defined as C50, C53, and C56, respectively [13]. Only cases for those within the age range of 20–79 were considered in this study, due to the fact that patients of breast, ovarian, and cervical cancer under 20 years old are very rare, and patients over 80 died from many other complicated causes; thus cases below 20 years old and above 80 years old were excluded in our study [14].

To perform APC analysis, the age-specific mortality rates were calculated for twelve different age groups (20–24, 25–29,…, 75–79) in three regions and twelve 5-year calendar periods (1954–1958, 1959–1963,…, 2009–2013) in Japan, while cases in Republic of Korea and Singapore were divided into five 5-year calendar periods (1989–1993, 1994–1998,…, 2009–2013) and ten calendar periods from 1964–1968 to 2009–2013, respectively. Furthermore, the studied periods on ovarian cancer in three countries of 1994–2013 were divided into four consecutive 5-year periods from 1994–1998 to 2009–2013. To characterize the mortality trends, age-standardized mortality rates (ASMRs) per 100,000 person-years were calculated by the direct method adjusted to the world standard population (1960) which is proposed by Segi [15] and modified by Doll et al. [16].

### 2.2. Statistical Analysis

Age, period, and cohort (APC) analysis is an important statistical tool broadly utilized in the fields of demography, sociology, and epidemiology in studying time-specific phenomenon [14, 17]. APC model is based on Poisson distribution which can reflect temporal trends of diseases in age, period, and cohort under the condition of adjustment for age, period, and cohort and explore the separate effects of chronological age, time period, and birth cohort [18]. Broadly defined, the age effects represent variation associated with different age groups and reflect the impact on event results of biology and sociology. The period effects represent the risk of variation over time that affect all age groups simultaneously. The cohort effects are associated with changes among groups of individuals born in the same year or years [17]. However, the well-known nonidentification problem still exists as there is a linear relationship between the age, period and cohort, that is,* cohort* =* period *−* age*, which makes it difficult to estimate the unique set for every age, period, and cohort effect [18]. To overcome this issue, a number of methodological contributions to the estimation of APC models have been introduced in different fields, including intrinsic estimator (IE), a new approach to the statistical estimation of age-period-cohort (APC) models, which has been developed recently [17]. Currently, the IE method has been widely utilized on disease incidence and mortality in many developed countries and regions [19]. In this study, the Akaike information criterion (AIC) and the Bayesian information criterion (BIC) were calculated to evaluate the goodness-of-fit of the model. The standard error (SE) of every model coefficient was calculated too. The STATA 12.0 software (Statacorp, College Station, TX, USA) was chosen as the statistical analysis software for all statistical analyses.

## 3. Results

**Figure 1:**
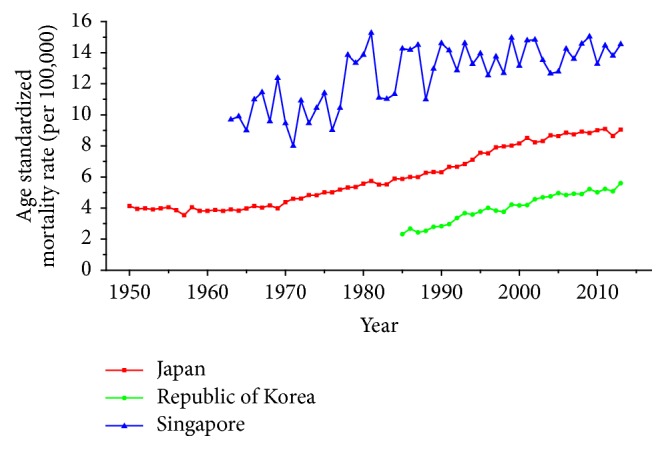
Trends of age-standardized mortality rates per 100,000 population for breast cancer in Japan, Republic of Korea, and Singapore.

**Figure 2:**
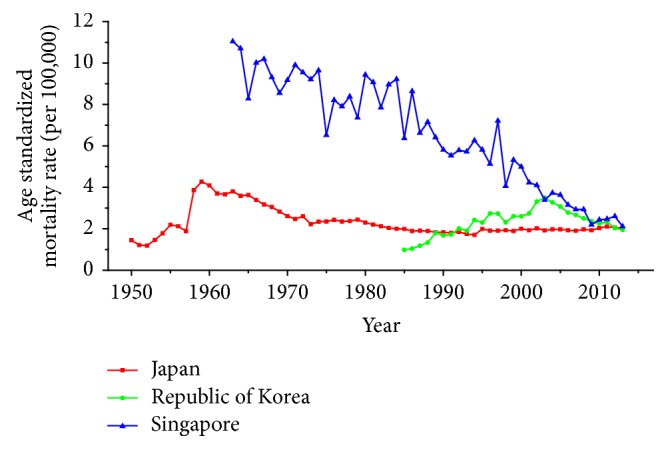
Trends of age-standardized mortality rates per 100,000 population for cervical cancer in Japan, Republic of Korea, and Singapore.

**Figure 3:**
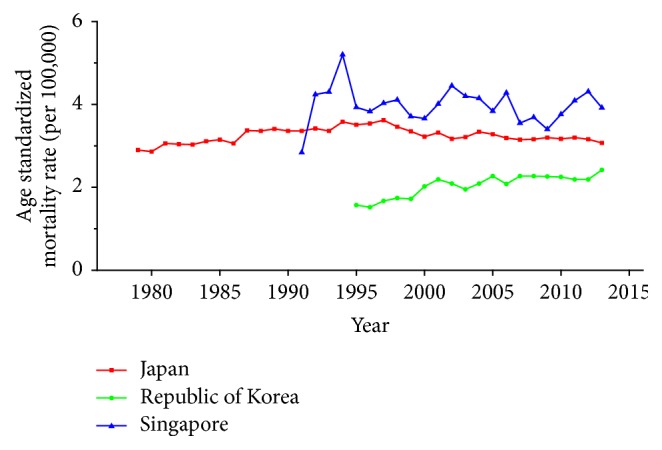
Trends of age-standardized mortality rates per 100,000 population for ovarian cancer in Japan, Republic of Korea, and Singapore.

Trends of age-standardized mortality rates (ASMRs) on breast cancer, cervical cancer, and ovarian cancer in Japan, Republic of Korea, and Singapore were shown from Figures 1–3 during the period of 1950–2013. For trends of breast cancer mortality rate, a general rising trend was presented in Figure 1 during the observation period in three countries. Among three regions during the whole period, Singapore had the highest ASMR, followed by Japan and Republic of Korea. Furthermore, the ASMRs in Japan and Republic of Korea showed a relatively stable upward trend whereas the ASMR in Singapore fluctuated significantly throughout the observation period. For trends of cervical cancer mortality rate in three countries, the change pattern in Singapore was different from the rest of the two countries which was presented in Figure 2; a fluctuated descending trend was observed in Singapore, from 11.04 to 2.11 per 100,000 female population. The ASMR in Japan generally showed an ascending trend before the year of 1959, followed by a significant decreasing trend after 1960 and this decline had leveled off since the year of 1973. The ASMR in Republic of Korea showed a bumpy upward trend before 2003 which was followed by a constant downward trend from 2003. Trends of ASMRs on ovarian cancer over time among three countries for the study period of 1979–2013 were shown in Figure 3. As is presented in Figure 3, the ASMRs in Japan and Republic of Korea showed a relatively stable trend with the exception of Singapore, which waved without obvious law during the observation periods. Additionally, Singapore had the highest mortality rate with the exception of 1991, whereas the ASMR in Republic of Korea maintained the lowest level throughout the observation period.

**Figure 4:**
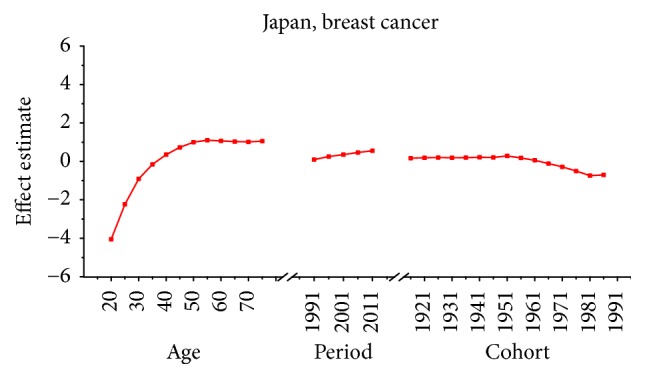
Age, period, and cohort effects of breast cancer mortality in Japan.

**Figure 5:**
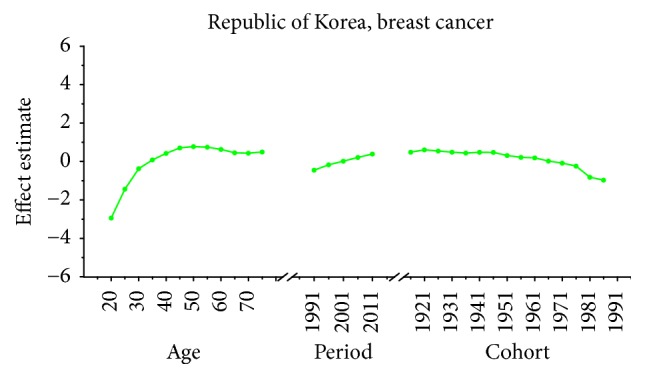
Age, period, and cohort effects of breast cancer mortality in Korea.

**Figure 6:**
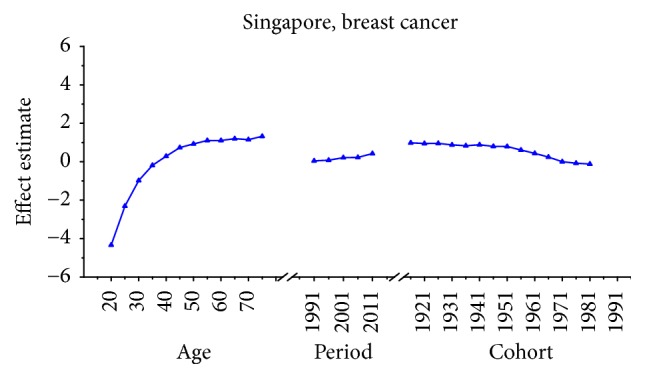
Age, period, and cohort effects of breast cancer mortality in Singapore.

**Figure 7:**
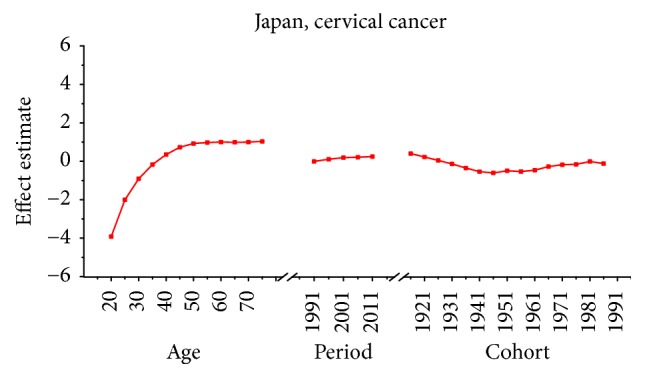
Age, period, and cohort effects of cervical cancer mortality in Japan.

**Figure 8:**
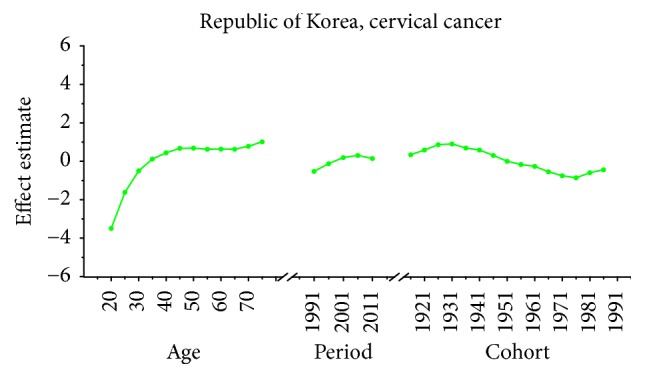
Age, period, and cohort effects of cervical cancer mortality in Korea.

**Figure 9:**
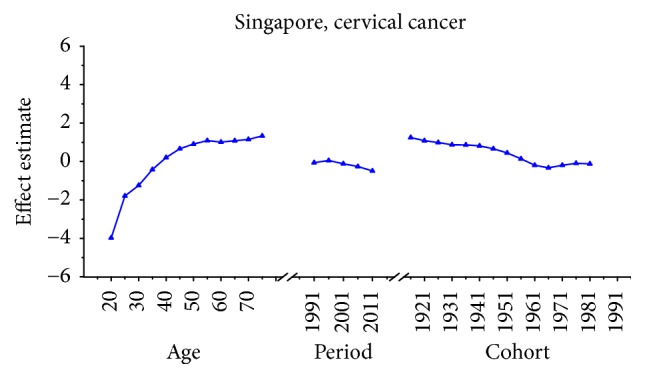
Age, period, and cohort effects of cervical cancer mortality in Singapore.

**Figure 10:**
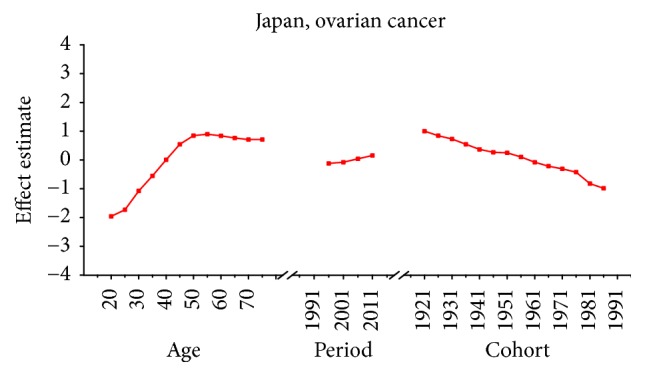
Age, period, and cohort effects of ovarian cancer mortality in Japan.

**Figure 11:**
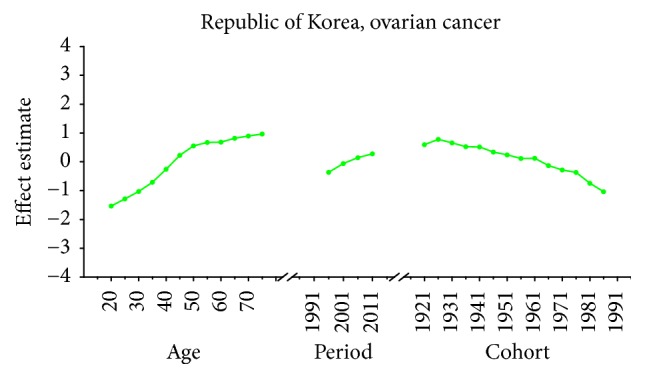
Age, period, and cohort effects of ovarian cancer mortality in Korea.

**Figure 12:**
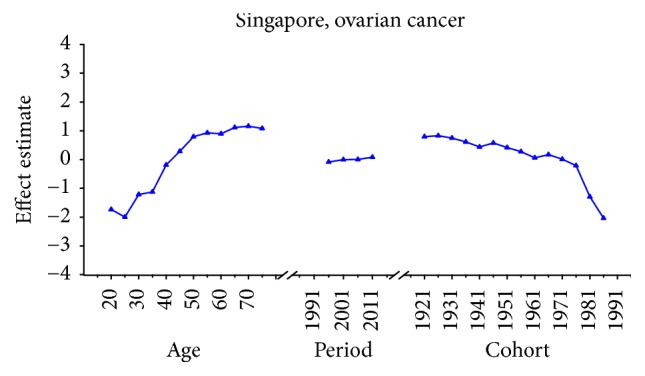
Age, period, and cohort effects of ovarian cancer mortality in Singapore.

Age-specific mortality rates for breast, cervical, and ovarian cancer by year of death in three countries are listed in Table S1–S9. The IE algorithm was used to calculate the age effect, period effect, and cohort effect. The results of APC model analysis are listed in Tables 1–3 for age-specific mortality on breast, cervical and ovarian cancer. Trends of age effect, period effect, and cohort effect on breast, ovarian, and cervical cancer in three countries are shown in Figures 4–12. The separate effect of each element was investigated as follows.

*Age Effect. *For breast cancer: the age effects increased continuously with age during the age group from 20 to 54 in all areas. Similar change pattern was observed in Japan and Republic of Korea that the effect plateaued at the age group 55–59 and began to decline gently after the age group 55–59. In contrast, age effect in Singapore increased constantly with the growth of age in all age groups from 20 to 79 and reached the bottom at group 75–79. For cervical cancer, the age effect presented an increasing trend with age from 20 to 54 in all areas whereas the effect decreased firstly and then increased from 60 to 79 in Japan and Republic of Korea. However, the coefficient of cervical cancer mortality estimation in Singapore generally increased with age with the exception of group 55–59 to 60–64; a slight decline was shown during this age periods. For ovarian cancer, age effect in Japan increased firstly and then decreased, reaching the peak at age group 55–59 whereas steady increasing trend was seen in Republic of Korea with the growth of age from 20 to 79. Notably, the effect in Singapore represented a volatile trend with age which peaked at the age group 70–74. Generally speaking, the age group 20–24 had the minimum coefficient of age effect estimation which indicated that the group of patients aged 20–24 years had the lowest cancer mortality in all areas.

*Period Effect. *For breast cancer: continuous increasing period effects were observed with time during the whole observation period in all three countries. For cervical cancer, the coefficient of mortality estimation in Japan and Republic of Korea generally increased with time while the effect in Singapore declined approximately during the whole study period. For ovarian cancer, the period effect showed a similar increasing trend to breast cancer during the observation period among all countries, which indicated an increasing mortality risk with time.

*Cohort Effect. *For breast cancer: the cohort effects showed different fluctuation trend among three countries. The cohort effect in Japan peaked at the birth cohort born in the 1950s and then began to decrease thereafter with a small reascending trend at the cohort born in 1982–1986. The effect in Republic of Korea increased for those who were born in 1912–1921 and began to decrease since 1920s, while a small reascending trend was observed for those born in 1932–1941. The effect in Singapore experienced two increases and two decreases with the generation born in 1912–1941 and then declined steadily thereafter. For cervical cancer, the cohort effects generally fluctuated unpredictably with the change of cohort year among all areas. The effect in Japan decreased first at the birth cohort born in 1912–1946 and then increased for the generation born in 1952–1981 again. For women in Republic of Korea, the effect decreased after the first increase and then ascended again, and it peaked with the birth cohort 1927–1931. However, the cohort effect in Singapore generally declined constantly with cohort year which waved violently during the generation born in 1982–1991. For ovarian cancer, similar variation trends were presented among three countries which generally decreased with cohort year. In particular, steady decrease of cohort effect was shown in Japan while some periods increased slightly in Republic of Korea and Singapore.

## 4. Discussion

This study has described the long-term variation tendency on three common female malignancies in three developed countries of Asia. To the best of our knowledge, APC analysis focused on cancer mortality trends has already been widely applied in many areas such as Japan [20], Korea [11], Taiwan [10], and USA [21]. However, a systematic comparison of the common malignant tumors using an age-period-cohort analysis in female populations among developed countries in Asia was scarcely reported before. The study by Lee et al. regarding the trends in gynecologic cancer mortality in East Asian regions has been published recently; however, they mainly focused on the comparison of uterine and ovarian cancer mortality trends using the Joinpoint Regression [22]. Thus, we conducted an age-period-cohort analysis via IE algorithm to evaluate the effects of three time-dependent parameters and assess the secular trends and patterns of age-specific breast, cervical, and ovarian cancer mortality among three developed countries of Asian female populations.

Our study has shown different trends of breast cancer mortality rates among three Asian countries. The ASMRs of breast cancer presented an overall upward trend in three areas whereas different degree and speed were shown among three counties as depicted in Figure 1. Our findings of mortality trends on breast cancer in three areas were consistent with the study by Wang et al. and Shin et al. [14, 23]. In some Western countries, however, converse trends of breast cancer mortality were reported, such as the study of Wang et al., which discovered the downward trend in the United States after 1990 [14]. Furthermore, in most individual European countries, similar decreasing trends were also observed in recent years [24]. The decreasing trend in European and some Western countries in recent years may attribute to the mammography screening, introduction of effective therapies, progress in surgery and radiotherapy, and improved medical intervention [23, 24].

The ASMRs of cervical cancer mortality also revealed different temporal patterns among three areas during the observation period. Although mortality of cervical cancer has decreased in some Western countries in recent decades, cervical cancer remained a major health problem in East Asia [25]. Although the declining tendency on cervical cancer mortality was observed in Japan since 1960s, the age-standardized incidence rate was still higher in Japan than in North America and the UK [26]. In Republic of Korea, the ASMR increased unevenly before 2003 which was followed by a gradual reduction trend thereafter. For women in Korea, the reduction of the overall cervical cancer incidence and mortality rates in Korea was mainly owing to the implementation of a population-based screening program since around 2000 [27]. Notably, although an apparent reduction of cervical cancer mortality was observed in Singapore, the ASMR in Singapore was still higher than that of Japan and Korea throughout the study periods.

The secular changes of ovarian cancer also yielded different temporal patterns for three counties. Although the burden of ovarian cancer in Japan is less dominant compared to other developed countries, the increasing trend of ASMR was still presented since 1979. The increase in Japan during this time may reflect the trend of increasing ovarian cancer incidence in the same population. Additionally, the change of dietary patterns, low fertility rate, and the low use of oral contraceptives might be also related to the increasing ovarian cancer rates in East Asia [22, 28]. In Korea, similar increasing trend of ovarian cancer mortality to Japan was shown, which may be explained for the reason of increased reporting from institutions and the under treatment of ovarian cancer; however, further studies were still needed to prove it [29]. For female population in Singapore, the ASMR of ovarian cancer peaked in 1994 and it was consistently much higher than the mortality in Japan and Korea from 1992 to 2013.

**Figure 13:**
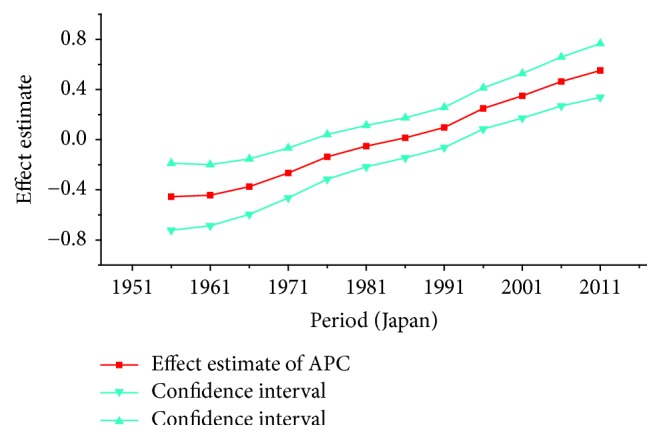
Period effect on breast cancer mortality from the results of APC-IE analysis in Japan.

**Figure 14:**
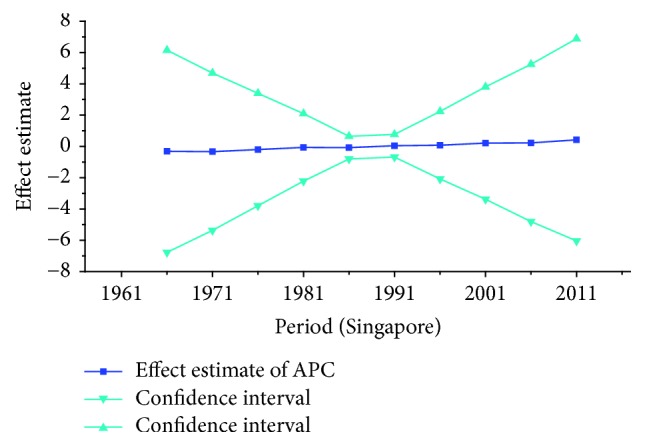
Period effect on breast cancer mortality from the results of APC-IE analysis in Singapore.

For breast cancer, the age effects showed clear difference among three Asian countries after menopause. For the women in Japan, the age effects were found to increase exponentially with age during the age groups from 20 to 54 and the effect plateaued at the age group 55–59. This phenomenon, in fact, was also observed in Republic of Korea and similar change pattern with the significant increase until age of 54 was presented while the effect also began to decline gently after the age group 55–59. The patterns of age effect during all age groups in Singapore, however, showed a gradual increase with age, which were different from those in Japan and Korea. These findings were consistent with the study of Shin et al., which described secular trends of breast cancer mortality in five East Asian populations [23]. The coefficient of breast cancer mortality estimation became positive (greater than 0) from 40 years old in all areas; this phenomenon might indicated the fact that age had become a contributing factor to the death of breast cancer for women over 40 years old [30]. Moreover, the minimum coefficient of age effect estimation in age group 20–24 indicated that this group was possibly at the lowest cancer mortality risk [19]. The discrepancies in various female populations among three areas may be affected by different levels of risk by birth cohort as well as differences in the treatment and the screening of the disease during different periods [23]. Our results of APC analysis (see in Table 1) showed that period effects in all three Asian areas increased throughout the study period, which was consistent with the ASMR trends in three counties (see in Figure 1). This phenomenon, however, might indicate that the period effects increased the risk of breast cancer mortality and it might be an important factor affecting the trend of breast cancer mortality. Although the reduction of breast cancer mortality risk was expected to be observed with the improvement of medical conditions and the implementation of the mammography screening, the increasing period effects were still presented which were probably affected by the environmental factors and dietary patterns [30]. Furthermore, we cannot identify the role of period effect due to the relatively short study period 1989–2013 in Korea; thus, a long-term data in Japan (1954–2013) and Singapore (1964–2013) was analyzed using APC-IE analysis, the results of which were shown in Figures 13 and 14. According to Figures 1 and 13, the period effect in Japan consistently increased from approximately 1960s and its mortality rate inclined simultaneously; the phenomenon was consistent with the study of Wang et al. [14]. Thus, we concluded that the period effect might be a more critical factor in the trend of breast cancer mortality than the other two effects. Overall, risk by birth cohort in all three countries showed a downward trend from the 1950s generation, which was similar to the findings of Wang et al. [14]. However, slight increase was still observed in some generations among three countries. The increased risk of breast cancer mortality may be mainly associated with the increased incidence rate of breast cancer, which is linked to an increase in prevalence of breast cancer risk factors. In Japan, the cohort effect peaked at the birth cohort born in 1947–1950 and then decreased thereafter with a small reascending trend at the cohort born in 1982–1986. It is considered that the changes in breast cancer risk factors in Asian countries can be largely attributed to rapid economic development [31]. In Republic of Korea, the effect increased for those who were born in 1912–1921 and began to decrease since the 1920s, except for a small reascending trend at birth cohort 1932–1941. The economic in Korea began to develop after the end of the Korean War in 1953, which was more rapid than the development in Japan; this time gap may be related to the different risk factors in two countries and contributed to the difference in birth years at the peak of mortality risk [32]. Usually, risk factors related to cohorts are associated with reproductive factors such as earlier age at menarche, late age at menopause, delayed age at first pregnancy, and escape from breastfeeding [33]. In Singapore, the effect experienced two increases and two decreases with the generation born in 1912–1941 and then declined steadily after the 1940s. Notably, family planning campaigns in Singapore from the 1960s also contributed to the decline in the total fertility of Singaporean women, which increased the cohort effect to a certain extent. Furthermore, a sharp declining trend was observed in the cohorts born in the 1990s, which may possibly be explained by rapid economic growth and social transformation in Singapore. In addition, the socioeconomic changes may also result in improved treatments and advances in screening, which also contributed to the sharply decreased cohort effects [22]. Additionally, dietary shifts towards fats as well as the trend towards increasing body mass index also could raise breast cancer risk among Asian women [34].

**Figure 15:**
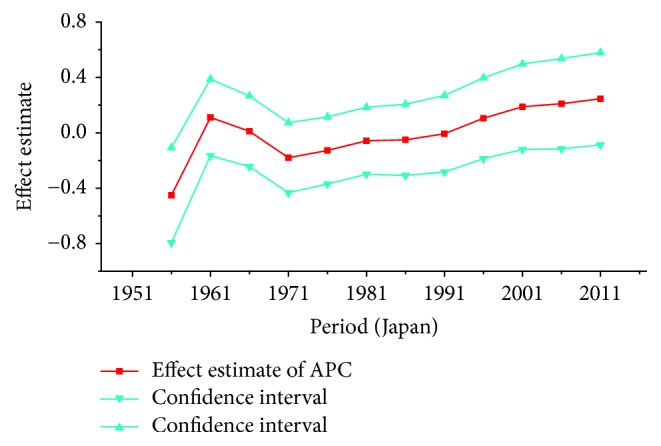
Period effect on cervical cancer mortality from the results of APC-IE analysis in Japan.

**Figure 16:**
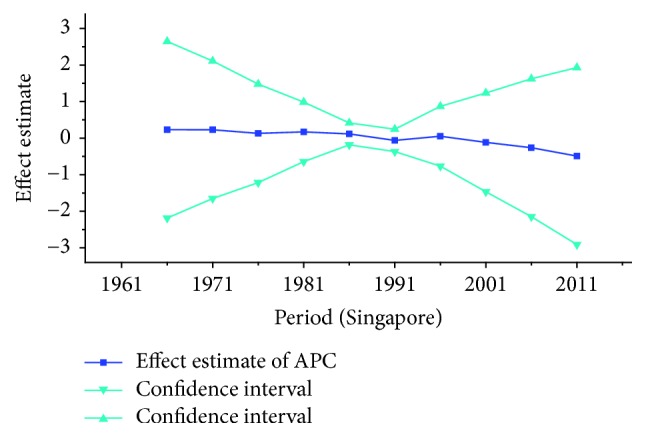
Period effect on cervical cancer mortality from the results of APC-IE analysis in Singapore.

For cervical cancer, the age effects presented an increasing trend with age from 20 to 54 in all three areas. For patients in Japan, the age effects revealed in the APC analysis were generally observed to increase significantly from 20 to 79 years, while a slight decrease was shown during the age group from 60–64 to 65–69. Similar to the trend of Japan, the age effect was generally found to increase with age from 20 to 79 years while two decreases were shown during the age group 50–54 to 55–59 and 60–64 to 65–69. Consistent with the other two countries, the age effect in Singapore was generally found to increase with age from 20 to 79 years with the exception of group 55–59 to 60–64; a slight decrease trend was observed during this age group. The overall trend of age effects among three counties might suggest that the older age became the higher mortality risk they would had, which might be explained for the reason that they might receive more exposure to low hygiene environments and had less opportunity to engage with preventive programs compared with younger age groups [27]. However, for women of middle-aged groups, the decreased age effects in three countries were possibly caused by the effective early detection and advanced diagnostic techniques [35]. For period effects among three countries, the APC analysis also yielded different change patterns during the whole study periods. For the effects in Japan and Republic of Korea, a general increasing trend was observed while the declining period effect in Singapore was shown during the whole periods. However, due to the relatively short period (1989–2013) in Korea, we could not exactly analyze the effect of period; thus, a long-term data in Japan (1954–2013) and Singapore (1964–2013) was analyzed using APC analysis to explore the period effect more accurately, the results of which were shown in Figures 15 and 16. According to Figure 15, the period effect in the Japan maintained the ascending trend before 1960 and began to decline significantly during the period from 1960 to 1970; this changing pattern, as presented in Figure 2, was consistent with the trend of ASMR on cervical cancer. For the period effect in Singapore as shown in Figure 16, a general declining trend was presented along with its mortality rate. Accordingly, we concluded that period effect might be an important factor affecting the trend of cervical cancer mortality via a series of clinic treatment and screen program. Generally speaking, the increasing period effects in Japan might be explained for the reason that screening and early detection programs were not successful as some countries in Europe [31], and the expected decreasing effect might be affected by strong cohort effects such as environmental factors and dietary patterns. However, with the improvement of medical conditions and the implementation of the cervical cancer screening, decreasing period effect was observed in Singapore. The cohort effects in three areas generally fluctuated inconsistently with the change of cohort year. For women in Japan, the cohort effects peaked with the cohort born in the 1900s and subsequently decreased; however, the effect increased again after the 1950s birth cohort, which was consistent with the study of Ito et al. [31]. Therefore, the decreased trend for cervical cancer mortality in Japan was probably due to the strong period effect with the implementation of a cervical cancer screening program since 1983 and small cohort effects related to the improvement of the public hygiene before the 1950s birth cohort [31]. For women in Republic of Korea, the cohort effect peaked at the cohort born in the 1930s and showed a general decreasing trend thereafter. For the younger generation in recent birth cohorts in Korea, a slight reascending effect was observed, which might be caused by the earlier onset of sexual activities that might cause early exposure to HPV infection. However, the declining trend of ASMR in cervical cancer was observed since 2003 which might be mainly caused by the period effect with the implementation of a population-based screening program since around 2000 and a decrease of cancer incidence. The reduction of cancer incidence was mainly caused by a decrease in the prevalence of cancer risk factors related to the improvements in public health policy and treatment of medical conditions that have occurred in recent cohort years [27]. For women in Singapore, the cohort effect peaked at the birth cohort born in the 1910s and declined slightly during the cohort period 1912–1981 while it dropped quickly in the younger cohorts during the generation born in 1982–1991. The sharp declining trend in the birth cohort 1990s was probably caused by rapid economic growth and social transformation in Singapore, and the consistently decreased trend of mortality might be driven by the period and cohort effect with improved treatments, increasing awareness of cervical cancer and advances in screening.

For ovarian cancer, the age effects showed a general increasing trend among three countries with the exception of Japan, which peaked at the age group 55–59 and began to decline after age of 60. The overall trend of age effects among three countries suggested that the advanced age might be an independent risk factor of ovarian cancer which might be possibly due to decreased performance status of older groups. Moreover, the increasing age effect in older women was probably due to the undertreatment of elderly patients [29]. For period effects among three countries, similar variation pattern was observed with an increasing trend throughout the study period, which indicated an increasing mortality risk with time. The increased mortality risk for ovarian cancer might indicate that there was little improvement of treatment and early detection in cancer diagnosis methods and no screening tests were introduced during the study period, which should have showed immediate effect for ovarian cancer [36]. Overall, the risk by birth cohort in all three countries peaked at the cohort born in the 1920s and began to decline thereafter. However, the increased trend of cohort effects was expected as they usually mirror trends in risk factors, and trends of risk factors would indicate an incline in risk for cancer mortality due to the cohort effect. Nevertheless, the real causes of the decrease in recent birth cohorts remain unclear which need further studies to confirm it. For women patients in Japan, the constant declining cohort effect was possibly due to some reproductive factors such as higher pregnancies or some components of the child bearing process which could protect patients by several mechanisms [28]. For patients in Korea, the general decreased cohort effect was probably related to an increasing parity among mothers of the babies born in boom years and oral contraceptive use and hysterectomy might also play an important role contributing to the decrease of cohort effects in younger generation [36]. However, a slight reascending effect for the cohort born in the 1920s and 1950s might be partly explained for the gradual westernization of lifestyles towards increased meat and fat consumption and changes in sociodemographic behavior factors which affected the environmental factors in Korea [37]. For cohort effect in Singapore, four slight increases were observed during different cohort groups in the 1920s, 1940s, 1960s, and late 1980s, respectively. The increased ASMR of ovarian cancer during the period 1991–2013 in Singapore might be explained as a result of increasing period effects, while small cohort effects were found for ovarian cancer in Singapore. Overall, the declining cohort effects for ovarian cancer mortality might be mainly related to the decrease of prevalence of cancer risk factors. The risk factors for ovarian cancer might be related to some changes in reproductive factors, such as nulliparity and a shift towards increased westernization of dietary patterns such as increased fat intake and talc use [36]. Furthermore, earlier age at menarche and later age at menopause might also increase the risk of ovarian cancer by increasing the number of ovulations [38].

Some specific limitations still exist in this study. A major limitation of the present study is the lack on incidence of breast, cervical, and ovarian cancer in three countries as the incidence also play a critical role affecting the mortality of cancer. Thus, further analysis combined with incidence is still needed in the future. Another limitation is the finite data of ovarian cancer mortality from WHO Mortality Database in three countries; the relatively short study periods may also result in imprecise estimations of the age-period-cohort effect on ovarian cancer. Therefore, all results from the APC analysis in this study still need further confirmation with more relevant studies. Furthermore, despite the nonbias, validity, asymptotic features, and superior estimation ability of the IE method, the parameter estimates generated using this method are not intuitive and the theory behind this method is complicated; therefore, further studies are still needed to explain the actual meaning of parameter estimates in the future.

## 5. Conclusions

In summary, we observed the general trends on common female malignances mortality rate in Japan, Republic of Korea, and Singapore from the period of 1954–2013, 1989–2013, and 1964–2013, respectively. Our study shows that the ASMRs of breast, cervical, and ovarian cancer maintain the highest level in Singapore compared to Japan and Korea. Generally speaking, the three cancer mortality rates in females increased with age which indicated that advanced age might be a risk factor. Moreover, the increased risk in younger generation was probably affected by reproductive factors and the shift towards westernized lifestyle and the dietary factors. Furthermore, period and cohort effects may collectively affect the common female malignances mortality for East Asian women, while period effects may still play an important role influencing the mortality of cervical cancer for Korea and Singapore.

## Figures and Tables

**Table 1:** APC model analysis results of breast cancer mortality in Japan, Republic of Korea, and Singapore.

	Japan		Republic of Korea		Singapore	
	Coef.	SE	Coef.	SE	Coef.	SE
*Age (year)*						
20–24	−4.0525	0.9292	−2.9416	1.2009	−4.3319	4.1333
25–29	−2.2338	0.3679	−1.4373	0.5212	−2.3113	3.3126
30–34	−0.9062	0.2184	−0.3749	0.3551	−0.9807	2.5717
35–39	−0.1572	0.1700	0.0834	0.2948	−0.1887	1.8381
40–44	0.3529	0.1438	0.4238	0.2530	0.2831	1.1071
45–49	0.7325	0.1261	0.7089	0.2157	0.7377	0.3845
50–54	0.9986	0.1138	0.7763	0.7763	0.9270	0.3810
55–59	1.1013	0.1068	0.7491	0.1659	1.1007	1.1029
60–64	1.0662	0.1043	0.6295	0.1557	1.1018	1.8335
65–69	1.0301	0.1045	0.4515	0.1598	1.1959	2.5652
70–74	1.0159	0.1064	0.4367	0.1712	1.1464	3.2974
75–79	1.0524	0.1104	0.4947	0.1972	1.3200	4.0298
*Period (year)*						
1991	0.0973	0.0816	−0.4497	0.1311	0.0459	0.3700
1996	0.2489	0.0837	−0.1713	0.1018	0.0779	1.1003
2001	0.3499	0.0907	0.0171	0.0902	0.2139	1.8324
2006	0.4637	0.0995	0.2135	0.0947	0.2242	2.5649
2011	0.5520	0.1093	0.3904	0.1104	0.4213	3.2974
*Cohort (year)*						
1912–1916	0.1649	0.2329	0.4849	0.4596	0.9832	58.2310
1917–1921	0.1907	0.2346	0.6056	0.3730	0.9447	58.9634
1922–1926	0.2027	0.2379	0.5468	0.3438	0.9499	59.6959
1927–1931	0.1888	0.2425	0.4849	0.3289	0.8740	60.4284
1932–1936	0.1929	0.2473	0.4425	0.3233	0.8313	61.1608
1937–1941	0.2117	0.2563	0.4783	0.3342	0.8816	61.8933
1942–1946	0.2038	0.2663	0.4758	0.3494	0.7952	62.6258
1947–1951	0.2822	0.2762	0.3094	0.3706	0.7927	63.3583
1952–1956	0.1786	0.2897	0.2152	0.3940	0.6065	64.0908
1957–1961	0.0532	0.3069	0.1882	0.4189	0.4344	64.8234
1962–1966	−0.1126	0.3317	0.0210	0.4504	0.2395	65.5559
1967–1971	−0.2816	0.3713	−0.0828	0.4856	−0.0017	66.2886
1972–1976	−0.5004	0.4523	−0.2374	0.5389	−0.0766	67.0214
1977–1981	−0.7393	0.6578	−0.8212	0.7075	−0.1215	67.7548
1982–1986	−0.7081	1.1861	−0.9692	1.1173	−0.3488	68.4933
1987–1991	−1.4416	4.8518	−2.1420	4.7930	−12.2362	1230.5410
Deviance	1.6419		0.6994		29.1817	
AIC	4.4232		4.5821		5.3158	
BIC	−495.3394		−122.1310		−353.8176	

**Table 2:** APC model analysis results of cervical cancer mortality in Japan, Republic of Korea, and Singapore.

	Japan		Republic of Korea		Singapore	
	Coef.	SE	Coef.	SE	Coef.	SE
*Age (year)*						
20–24	−3.9142	1.3332	−3.4937	2.0312	−3.9745	1.8634
25–29	−2.0033	0.5229	−1.6183	0.8333	−1.7915	1.2805
30–34	−0.9070	0.3411	−0.4979	0.5947	−1.2451	0.9979
35–39	−0.1740	0.2672	0.1162	0.5029	−0.4178	0.7154
40–44	0.3498	0.2256	0.4420	0.4315	0.2071	0.4453
45–49	0.7281	0.1976	0.6746	0.3635	0.6627	0.2019
50–54	0.9255	0.1782	0.6856	0.3050	0.9078	0.1885
55–59	0.9751	0.1643	0.6305	0.2531	1.0838	0.4254
60–64	1.0033	0.1531	0.6354	0.2080	1.0106	0.6907
65–69	0.9852	0.1464	0.6316	0.1872	1.0806	0.9605
70–74	0.9978	0.1425	0.7776	0.1957	1.1458	1.2319
75–79	1.0337	0.1424	1.0164	0.2357	1.3305	1.5042
*Period (year)*						
1991	−0.0069	0.1412	−0.5241	0.1904	−0.0619	0.1562
1996	0.1052	0.1490	−0.1242	0.1309	0.0523	0.4174
2001	0.1889	0.1578	0.1925	0.1025	−0.1159	0.6892
2006	0.2100	0.1666	0.3089	0.1265	−0.2612	0.9619
2011	0.2463	0.1700	0.1470	0.1738	−0.4907	1.2350
*Cohort (year)*						
1912–1916	0.3999	0.2172	0.3426	0.5182	1.2454	21.5898
1917–1921	0.2212	0.2262	0.5863	0.3970	1.0811	21.8614
1922–1926	0.0452	0.2369	0.8657	0.3327	0.9869	22.1330
1927–1931	−0.1394	0.2490	0.9048	0.3034	0.8695	22.4047
1932–1936	−0.3492	0.2612	0.6902	0.3011	0.8649	22.6764
1937–1941	−0.5377	0.2863	0.5823	0.3270	0.8111	22.9482
1942–1946	−0.6053	0.3085	0.3043	0.3692	0.6621	23.2201
1947–1951	−0.4931	0.3227	−0.0015	0.4209	0.4439	23.4920
1952–1956	−0.5321	0.3486	−0.1655	0.4728	0.1389	23.7642
1957–1961	−0.4611	0.3732	−0.2699	0.5244	−0.1924	24.0367
1962–1966	−0.2788	0.3994	−0.5451	0.5903	−0.3294	24.3097
1967–1971	−0.1807	0.4498	−0.7526	0.6697	−0.1936	24.5829
1972–1976	−0.1622	0.5504	−0.8588	0.7769	−0.0947	24.8579
1977–1981	−0.0086	0.7250	−0.5983	0.9026	−0.1206	25.1393
1982–1986	−0.1180	1.3189	−0.4424	1.3826	−0.7370	25.4727
1987–1991	−0.1738	4.1150	−0.6419	4.6053	−9.0625	456.2361
Deviance	1.7567		1.1981		30.4706	
AIC	3.6218		4.0739		4.6238	
BIC	−495.2246		−121.6323		−352.5287	

**Table 3:** APC model analysis results of ovarian cancer mortality in Japan, Republic of Korea, and Singapore.

	Japan		Republic of Korea		Singapore	
	Coef.	SE	Coef.	SE	Coef.	SE
*Age (year)*						
20–24	−1.9564	1.0350	−1.5340	1.0734	−1.7342	1.0433
25–29	−1.7304	0.7743	−1.2848	0.7817	−1.9964	0.8930
30–34	−1.0773	0.5786	−1.0276	0.6637	−1.2136	0.5904
35–39	−0.5521	0.4695	−0.7083	0.5653	−1.1246	0.5256
40–44	0.0071	0.3947	−0.2547	0.4909	−0.1881	0.3996
45–49	0.5464	0.3210	0.2250	0.4083	0.2877	0.3255
50–54	0.8483	0.2594	0.5542	0.3334	0.7948	0.2544
55–59	0.8943	0.2112	0.6709	0.2726	0.9271	0.2095
60–64	0.8378	0.1815	0.6816	0.2288	0.8949	0.2010
65–69	0.7640	0.1848	0.8176	0.2126	1.1125	0.2264
70–74	0.7087	0.2197	0.8945	0.2438	1.1595	0.2873
75–79	0.7095	0.2821	0.9657	0.3147	1.0805	0.3674
*Period (year)*						
1996	−0.1198	0.1432	−0.3632	0.1814	−0.0857	0.1559
2001	−0.0775	0.0997	−0.0601	0.1244	−0.0019	0.0919
2006	0.0411	0.1023	0.1464	0.1229	0.0051	0.0955
2011	0.1562	0.1379	0.2769	0.1637	0.0825	0.1533
*Cohort (year)*						
1917–1921	1.0013	0.3983	0.5938	0.4961	0.7966	0.4138
1922–1926	0.8437	0.3054	0.7791	0.3525	0.8288	0.3086
1927–1931	0.7276	0.2455	0.6567	0.2772	0.7477	0.2393
1932–1936	0.5444	0.2117	0.5252	0.2318	0.6114	0.2004
1937–1941	0.3670	0.2249	0.5175	0.2460	0.4375	0.2156
1942–1946	0.2687	0.2598	0.3379	0.2995	0.5753	0.2588
1947–1951	0.2499	0.3073	0.2407	0.3660	0.4153	0.3299
1952–1956	0.1024	0.3674	0.1146	0.4386	0.2764	0.4057
1957–1961	−0.0768	0.4321	0.1242	0.5042	0.0604	0.4930
1962–1966	−0.2156	0.4996	−0.1301	0.5860	0.1700	0.5653
1967–1971	−0.3090	0.5692	−0.2827	0.6529	0.0129	0.6539
1972–1976	−0.4221	0.6297	−0.3669	0.6901	−0.2111	0.7210
1977–1981	−0.8206	0.8884	−0.7438	0.9024	−1.2924	1.1975
1982–1986	−0.9827	1.2780	−1.0378	1.2507	−2.0408	2.1854
1987–1991	−1.2783	2.4498	−1.3283	2.4048	−1.3881	2.4715
Deviance	0.0872		0.4661		4.6688	
AIC	4.4618		4.0862		4.6186	
BIC	−77.3368		−76.9580		−72.7553	

## Supplementary Materials

Supplementary Materials The supplementary materials from Tables S1 to S9 describe the age-specific mortality rates of breast cancer, cervical cancer, and ovarian cancer in three countries. Table S1: age-specific mortality rates (per 100,000 women) of breast cancer in Japan. Table S2: age-specific mortality rates (per 100,000 women) of breast cancer in Singapore. Table S3: age-specific mortality rates (per 100,000 women) of breast cancer in Republic of Korea. Table S4: age-specific mortality rates (per 100,000 women) of cervical cancer in Japan. Table S5: age-specific mortality rates (per 100,000 women) of cervical cancer in Singapore. Table S6: age-specific mortality rates (per 100,000 women) of cervical cancer in Republic of Korea. Table S7: age-specific mortality rates (per 100,000 women) of ovarian cancer in Japan. Table S8: age-specific mortality rates (per 100,000 women) of ovarian cancer in Singapore. Table S9: age-specific mortality rates (per 100,000 women) of ovarian cancer in Republic of Korea.
