# Supplementary material for: Temporal Trends of Common Female Malignances on Breast, Cervical, and Ovarian Cancer Mortality in Japan, Republic of Korea, and Singapore: Application of the Age-Period-Cohort Model
Source: Biomed Res Int. 2018 Mar 21;2018:5307459. doi: 10.1155/2018/5307459 (PMC5884400; doi:10.1155/2018/5307459)
Supplement: Supplementary Materials — The supplementary materials from Tables S1 to S9 describe the age-specific mortality rates of breast cancer, cervical cancer, and ovarian cancer in three countries. Table S1: age-specific mortality rates (per 100,000 women) of breast cancer in Japan. Table S2: age-specific mortality rates (per 100,000 women) of breast cancer in Singapore. Table S3: age-specific mortality rates (per 100,000 women) of breast cancer in Republic of Korea. Table S4: age-specific mortality rates (per 100,000 women) of cervical cancer in Japan. Table S5: age-specific mortality rates (per 100,000 women) of cervical cancer in Singapore. Table S6: age-specific mortality rates (per 100,000 women) of cervical cancer in Republic of Korea. Table S7: age-specific mortality rates (per 100,000 women) of ovarian cancer in Japan. Table S8: age-specific mortality rates (per 100,000 women) of ovarian cancer in Singapore. Table S9: age-specific mortality rates (per 100,000 women) of ovarian cancer in Republic of Korea. [file 5307459.f1.docx]

**Table S1.** Age-specific mortality rates (per 100, 000 women) of breast cancer in Japan

| **Age** | **Year of Death** | | | | | | | | | | | |
| --- | --- | --- | --- | --- | --- | --- | --- | --- | --- | --- | --- | --- |
|  | 1954-1958 | 1959-1963 | 1964-1968 | 1969-1973 | 1974-1978 | 1979-1983 | 1984-1988 | 1989-1993 | 1994-1998 | 1999-2003 | 2004-2008 | 2009-2013 |
| 20-24 | 0.06 | 0.07 | 0.05 | 0.11 | 0.09 | 0.09 | 0.14 | 0.05 | 0.08 | 0.08 | 0.10 | 0.04 |
| 25-29 | 0.47 | 0.40 | 0.48 | 0.47 | 0.67 | 0.81 | 0.74 | 0.59 | 0.57 | 0.51 | 0.48 | 0.49 |
| 30-34 | 1.75 | 1.64 | 1.79 | 2.20 | 2.44 | 2.73 | 2.71 | 2.80 | 2.57 | 2.58 | 2.24 | 1.84 |
| 35-39 | 4.11 | 4.07 | 3.96 | 4.47 | 4.75 | 5.03 | 5.87 | 6.37 | 7.02 | 6.40 | 5.68 | 4.99 |
| 40-44 | 6.68 | 6.88 | 6.91 | 7.55 | 8.15 | 8.72 | 9.46 | 11.36 | 12.23 | 11.90 | 11.38 | 10.41 |
| 45-49 | 8.82 | 9.44 | 9.97 | 11.06 | 12.61 | 13.00 | 14.41 | 15.34 | 19.56 | 20.09 | 19.37 | 17.70 |
| 50-54 | 10.38 | 10.78 | 12.51 | 14.06 | 16.53 | 17.12 | 18.58 | 20.97 | 24.85 | 29.23 | 28.45 | 27.30 |
| 55-59 | 11.81 | 11.48 | 12.93 | 14.26 | 17.14 | 20.05 | 21.54 | 23.37 | 26.48 | 29.56 | 36.24 | 35.05 |
| 60-64 | 12.48 | 11.37 | 12.17 | 14.10 | 16.27 | 18.92 | 20.74 | 21.88 | 25.55 | 29.10 | 31.88 | 37.49 |
| 65-69 | 13.34 | 12.23 | 12.38 | 13.21 | 16.14 | 17.84 | 18.86 | 21.14 | 24.60 | 26.79 | 31.27 | 33.69 |
| 70-74 | 15.42 | 14.43 | 13.34 | 13.78 | 14.77 | 17.53 | 18.98 | 20.68 | 23.39 | 25.47 | 29.50 | 33.51 |
| 75-79 | 17.71 | 15.85 | 15.36 | 16.24 | 16.88 | 17.11 | 18.63 | 20.14 | 24.44 | 27.07 | 30.51 | 34.38 |

**Table S2.** Age-specific mortality rates (per 100, 000 women) of breast cancer in Singapore

| **Age** | **Year of Death** | | | | | | | | | |
| --- | --- | --- | --- | --- | --- | --- | --- | --- | --- | --- |
|  | 1964-1968 | 1969-1973 | 1974-1978 | 1979-1983 | 1984-1988 | 1989-1993 | 1994-1998 | 1999-2003 | 2004-2008 | 2009-2013 |
| 20-24 | 0.00 | 0.00 | 0.31 | 0.14 | 0.29 | 0.00 | 0.17 | 0.00 | 0.00 | 0.00 |
| 25-29 | 0.30 | 1.11 | 0.36 | 1.56 | 1.40 | 1.41 | 0.15 | 0.73 | 0.75 | 0.72 |
| 30-34 | 4.55 | 4.35 | 3.59 | 5.06 | 4.96 | 2.86 | 2.99 | 2.42 | 2.08 | 3.09 |
| 35-39 | 6.12 | 6.39 | 9.50 | 10.66 | 14.01 | 9.49 | 8.56 | 8.23 | 6.89 | 7.56 |
| 40-44 | 12.84 | 14.04 | 12.85 | 15.77 | 17.40 | 22.77 | 16.58 | 15.33 | 14.40 | 12.70 |
| 45-49 | 28.11 | 25.75 | 26.05 | 24.83 | 32.59 | 29.14 | 28.51 | 27.63 | 21.96 | 22.17 |
| 50-54 | 26.75 | 26.83 | 29.60 | 39.35 | 33.14 | 42.07 | 34.18 | 38.56 | 38.35 | 39.13 |
| 55-59 | 42.08 | 38.94 | 32.80 | 43.08 | 42.06 | 42.90 | 45.36 | 49.18 | 52.72 | 47.32 |
| 60-64 | 32.51 | 27.52 | 43.65 | 47.77 | 39.82 | 42.64 | 47.86 | 58.82 | 55.41 | 57.89 |
| 65-69 | 38.25 | 36.39 | 42.08 | 51.12 | 49.15 | 57.37 | 53.73 | 53.92 | 57.52 | 67.09 |
| 70-74 | 33.42 | 33.27 | 46.40 | 48.86 | 44.92 | 48.90 | 55.27 | 58.18 | 53.78 | 68.52 |
| 75-79 | 34.14 | 45.91 | 48.60 | 47.36 | 46.32 | 61.84 | 64.71 | 77.32 | 64.36 | 89.08 |

**Table S3.** Age-specific mortality rates (per 100, 000 women) of breast cancer in Republic of Korea

| **Age** | **Year of Death** | | | | |
| --- | --- | --- | --- | --- | --- |
|  | 1989-1993 | 1994-1998 | 1999-2003 | 2004-2008 | 2009-2013 |
| 20-24 | 0.10 | 0.19 | 0.11 | 0.10 | 0.04 |
| 25-29 | 0.78 | 0.88 | 0.76 | 0.45 | 0.59 |
| 30-34 | 2.24 | 2.63 | 2.85 | 2.81 | 2.06 |
| 35-39 | 3.72 | 4.70 | 4.83 | 5.52 | 5.69 |
| 40-44 | 6.14 | 6.87 | 7.88 | 8.70 | 8.89 |
| 45-49 | 9.73 | 9.61 | 11.29 | 13.44 | 13.08 |
| 50-54 | 10.63 | 12.19 | 12.96 | 14.26 | 17.27 |
| 55-59 | 8.89 | 12.41 | 15.25 | 15.53 | 17.29 |
| 60-64 | 8.23 | 10.65 | 13.60 | 16.05 | 17.00 |
| 65-69 | 6.96 | 9.72 | 10.89 | 13.73 | 16.53 |
| 70-74 | 7.26 | 10.45 | 11.43 | 13.68 | 15.23 |
| 75-79 | 7.42 | 11.70 | 12.58 | 14.07 | 16.18 |

**Table S4.** Age-specific mortality rates (per 100, 000 women) of cervical cancer in Japan

| **Age** | **Year of Death** | | | | | | | | | | | |
| --- | --- | --- | --- | --- | --- | --- | --- | --- | --- | --- | --- | --- |
|  | 1954-1958 | 1959-1963 | 1964-1968 | 1969-1973 | 1974-1978 | 1979-1983 | 1984-1988 | 1989-1993 | 1994-1998 | 1999-2003 | 2004-2008 | 2009-2013 |
| 20-24 | 0.05 | 0.04 | 0.03 | 0.03 | 0.02 | 0.03 | 0.05 | 0.06 | 0.02 | 0.05 | 0.07 | 0.06 |
| 25-29 | 0.23 | 0.27 | 0.29 | 0.16 | 0.14 | 0.14 | 0.17 | 0.32 | 0.41 | 0.44 | 0.46 | 0.42 |
| 30-34 | 0.66 | 1.02 | 0.91 | 0.54 | 0.45 | 0.53 | 0.64 | 0.63 | 1.11 | 1.36 | 1.14 | 1.45 |
| 35-39 | 1.75 | 2.73 | 2.25 | 1.57 | 1.16 | 0.97 | 1.19 | 1.18 | 1.71 | 2.41 | 2.49 | 2.58 |
| 40-44 | 4.1 | 5.49 | 4.07 | 3.32 | 2.59 | 2.05 | 1.9 | 2.25 | 2.62 | 3.08 | 3.71 | 3.87 |
| 45-49 | 6.78 | 8.99 | 6.97 | 5.5 | 4.88 | 3.85 | 3.16 | 3.15 | 3.77 | 4.26 | 4.59 | 5.13 |
| 50-54 | 7.91 | 12.38 | 10.42 | 7.55 | 6.84 | 5.86 | 4.5 | 4.16 | 4.46 | 5.46 | 5.26 | 5.62 |
| 55-59 | 7.99 | 14.32 | 12.34 | 9.27 | 8.07 | 7.32 | 6.19 | 5.12 | 5.06 | 5.23 | 5.94 | 5.5 |
| 60-64 | 8.51 | 15.62 | 14.18 | 10.49 | 9.74 | 9.02 | 7.38 | 6.37 | 5.83 | 5.17 | 4.99 | 6.02 |
| 65-69 | 8.06 | 14.35 | 14.07 | 10.53 | 10.9 | 10.84 | 8.88 | 8.07 | 7.1 | 5.99 | 5.21 | 5.35 |
| 70-74 | 6.79 | 15.17 | 13.13 | 11.26 | 11.09 | 12.66 | 11.12 | 9.55 | 8.36 | 7.41 | 6.52 | 5.93 |
| 75-79 | 6.35 | 13.95 | 12.73 | 10.57 | 12.81 | 12.38 | 13.02 | 11.79 | 11.08 | 9.77 | 8.22 | 7.6 |

**Table S5.** Age-specific mortality rates (per 100, 000 women) of cervical cancer in Singapore

| **Age** | **Year of Death** | | | | | | | | | |
| --- | --- | --- | --- | --- | --- | --- | --- | --- | --- | --- |
|  | 1964-1968 | 1969-1973 | 1974-1978 | 1979-1983 | 1984-1988 | 1989-1993 | 1994-1998 | 1999-2003 | 2004-2008 | 2009-2013 |
| 20-24 | 0.00 | 0.00 | 0.00 | 0.28 | 0.00 | 0.17 | 0.00 | 0.00 | 0.18 | 0.00 |
| 25-29 | 1.20 | 2.57 | 0.36 | 1.11 | 0.28 | 0.42 | 0.29 | 0.44 | 0.15 | 0.00 |
| 30-34 | 3.51 | 2.60 | 0.92 | 2.34 | 0.63 | 0.67 | 1.04 | 0.40 | 0.77 | 0.77 |
| 35-39 | 7.32 | 5.70 | 4.40 | 5.40 | 2.98 | 3.07 | 2.22 | 1.37 | 1.27 | 1.12 |
| 40-44 | 15.69 | 8.60 | 7.13 | 11.80 | 10.04 | 8.83 | 4.92 | 2.06 | 1.61 | 2.42 |
| 45-49 | 29.63 | 21.07 | 16.83 | 16.30 | 13.75 | 9.76 | 9.37 | 5.95 | 4.11 | 3.00 |
| 50-54 | 34.79 | 23.86 | 22.33 | 26.32 | 25.31 | 16.70 | 15.54 | 12.12 | 7.29 | 4.45 |
| 55-59 | 34.31 | 42.18 | 37.78 | 26.98 | 25.61 | 21.63 | 22.16 | 16.43 | 9.82 | 6.36 |
| 60-64 | 25.88 | 32.36 | 35.57 | 31.35 | 25.69 | 22.41 | 23.54 | 17.81 | 14.50 | 7.85 |
| 65-69 | 30.46 | 35.94 | 29.90 | 40.26 | 33.72 | 22.42 | 18.78 | 21.55 | 16.26 | 10.70 |
| 70-74 | 43.10 | 30.67 | 35.12 | 35.44 | 34.96 | 21.90 | 36.70 | 22.82 | 19.18 | 17.21 |
| 75-79 | 7.84 | 63.91 | 34.77 | 35.53 | 41.66 | 42.86 | 35.39 | 27.18 | 22.73 | 15.00 |

**Table S6.** Age-specific mortality rates (per 100, 000 women) of cervical cancer in Republic of Korea

| **Age** | **Year of Death** | | | | |
| --- | --- | --- | --- | --- | --- |
|  | 1989-1993 | 1994-1998 | 1999-2003 | 2004-2008 | 2009-2013 |
| 20-24 | 0.04 | 0.02 | 0.04 | 0.08 | 0.05 |
| 25-29 | 0.23 | 0.22 | 0.34 | 0.30 | 0.39 |
| 30-34 | 0.75 | 0.95 | 0.75 | 0.90 | 1.16 |
| 35-39 | 1.67 | 2.29 | 1.95 | 1.74 | 1.48 |
| 40-44 | 2.59 | 3.28 | 3.63 | 2.95 | 2.67 |
| 45-49 | 4.28 | 5.20 | 5.03 | 4.98 | 3.95 |
| 50-54 | 5.42 | 6.71 | 6.04 | 6.06 | 5.27 |
| 55-59 | 5.33 | 8.38 | 8.49 | 6.78 | 5.23 |
| 60-64 | 7.96 | 8.90 | 10.81 | 9.30 | 5.82 |
| 65-69 | 6.65 | 10.71 | 12.88 | 13.00 | 7.60 |
| 70-74 | 6.80 | 11.66 | 17.35 | 16.80 | 11.70 |
| 75-79 | 6.22 | 11.29 | 22.65 | 25.22 | 16.50 |

**Table S7.** Age-specific mortality rates (per 100, 000 women) of ovarian cancer in Japan

| **Age** | **Year of Death** | | | |
| --- | --- | --- | --- | --- |
|  | 1994-1998 | 1999-2003 | 2004-2008 | 2009-2013 |
| 20-24 | 0.29 | 0.22 | 0.21 | 0.17 |
| 25-29 | 0.41 | 0.44 | 0.28 | 0.28 |
| 30-34 | 0.94 | 0.83 | 0.83 | 0.66 |
| 35-39 | 1.75 | 1.56 | 1.65 | 1.63 |
| 40-44 | 3.56 | 3.32 | 3.07 | 3.21 |
| 45-49 | 7.51 | 6.34 | 6.07 | 6.05 |
| 50-54 | 9.90 | 10.18 | 10.19 | 9.29 |
| 55-59 | 11.56 | 11.10 | 11.86 | 11.76 |
| 60-64 | 12.69 | 11.49 | 11.86 | 12.86 |
| 65-69 | 14.81 | 12.64 | 11.96 | 11.85 |
| 70-74 | 15.54 | 14.33 | 13.57 | 12.54 |
| 75-79 | 18.13 | 16.09 | 15.99 | 15.40 |

**Table S8.** Age-specific mortality rates (per 100, 000 women) of ovarian cancer in Singapore

| **Age** | **Year of Death** | | | |
| --- | --- | --- | --- | --- |
|  | 1994-1998 | 1999-2003 | 2004-2008 | 2009-2013 |
| 20-24 | 0.53 | 0.19 | 0.00 | 0.17 |
| 25-29 | 0.29 | 0.30 | 0.30 | 0.15 |
| 30-34 | 1.17 | 0.93 | 1.16 | 0.13 |
| 35-39 | 0.92 | 1.62 | 1.40 | 0.74 |
| 40-44 | 3.70 | 2.72 | 3.70 | 3.31 |
| 45-49 | 7.00 | 5.70 | 5.65 | 5.63 |
| 50-54 | 12.88 | 12.24 | 10.02 | 9.09 |
| 55-59 | 13.48 | 16.27 | 11.82 | 13.66 |
| 60-64 | 16.31 | 11.66 | 14.62 | 15.40 |
| 65-69 | 22.29 | 17.59 | 17.25 | 21.40 |
| 70-74 | 19.85 | 24.20 | 23.99 | 19.70 |
| 75-79 | 21.31 | 27.90 | 20.57 | 18.64 |

**Table S9.** Age-specific mortality rates (per 100, 000 women) of ovarian cancer in Republic of Korea

| **Age** | **Year of Death** | | | |
| --- | --- | --- | --- | --- |
|  | 1994-1998 | 1999-2003 | 2004-2008 | 2009-2013 |
| 20-24 | 0.30 | 0.24 | 0.15 | 0.18 |
| 25-29 | 0.35 | 0.35 | 0.38 | 0.37 |
| 30-34 | 0.61 | 0.53 | 0.70 | 0.51 |
| 35-39 | 1.05 | 0.91 | 0.95 | 1.09 |
| 40-44 | 1.61 | 1.82 | 1.72 | 1.99 |
| 45-49 | 2.77 | 2.93 | 3.89 | 3.60 |
| 50-54 | 4.35 | 4.66 | 5.36 | 6.27 |
| 55-59 | 5.24 | 6.55 | 6.61 | 7.01 |
| 60-64 | 4.96 | 7.80 | 7.45 | 8.39 |
| 65-69 | 7.15 | 8.93 | 10.76 | 9.54 |
| 70-74 | 8.91 | 10.54 | 11.83 | 12.55 |
| 75-79 | 7.90 | 12.82 | 14.18 | 13.87 |
